# Tailoring the size of ultrasound responsive lipid-shelled nanodroplets by varying production parameters and environmental conditions

**DOI:** 10.1016/j.ultsonch.2021.105482

**Published:** 2021-02-03

**Authors:** Sara Ferri, Qiang Wu, Antonio De Grazia, Anastasia Polydorou, Jonathan P. May, Eleanor Stride, Nicholas D. Evans, Dario Carugo

**Affiliations:** a Faculty of Engineering and Physical Sciences, Department of Mechanical Engineering, University of Southampton, UK; b Centre for Human Development, Stem Cells and Regeneration, Bioengineering Sciences, Faculty of Medicine, University of Southampton, UK; c Department of Engineering Science, University of Oxford, UK; d Institute for Life Sciences (IfLS), University of Southampton, UK; e Department of Pharmaceutics, School of Pharmacy, University College London (UCL), UK

**Keywords:** Perfluorocarbon nanodroplet, Ultrasound, Drug delivery, Nanodroplet stability, Microbubble, Acoustic droplet vaporisation

## Abstract

Liquid perfluorocarbon nanodroplets (NDs) are an attractive alternative to microbubbles (MBs) for ultrasound-mediated therapeutic and diagnostic applications. ND size and size distribution have a strong influence on their behaviour *in vivo*, including extravasation efficiency, circulation time, and response to ultrasound stimulation. Thus, it is desirable to identify ways to tailor the ND size and size distribution during manufacturing. In this study phospholipid-coated NDs, comprising a perfluoro-n-pentane (PFP) core stabilised by a DSPC/PEG40s (1,2-distearoyl-*sn*-glycero-3-phosphocholine and polyoxyethylene(40)stearate, 9:1 molar ratio) shell, were produced in phosphate-buffered saline (PBS) by sonication. The effect of the following production-related parameters on ND size was investigated: PFP concentration, power and duration of sonication, and incorporation of a lipophilic fluorescent dye. ND stability was also assessed at both 4 °C and 37 °C. When a sonication pulse of 6 s and 15% duty cycle was employed, increasing the volumetric concentration of PFP from 5% to 15% v/v in PBS resulted in an increase in ND diameter from 215.8 ± 16.8 nm to 408.9 ± 171.2 nm. An increase in the intensity of sonication from 48 to 72 W (with 10% PFP v/v in PBS) led to a decrease in ND size from 354.6 ± 127.2 nm to 315.0 ± 100.5 nm. Increasing the sonication time from 20 s to 40 s (using a pulsed sonication with 30% duty cycle) did not result in a significant change in ND size (in the range 278–314 nm); however, when it was increased to 60 s, the average ND diameter reduced to 249.7 ± 9.7 nm, which also presented a significantly lower standard deviation compared to the other experimental conditions investigated (i.e., 9.7 nm *vs.* > 49.4 nm). The addition of the fluorescent dye DiI at different molar ratios did not affect the ND size distribution. NDs were stable at 4 °C for up to 6 days and at 37 °C for up to 110 min; however, some evidence of ND-to-MB phase transition was observed after 40 min at 37 °C. Finally, phase transition of NDs into MBs was demonstrated using a tissue-mimicking flow phantom under therapeutic ultrasound exposure conditions (ultrasound frequency: 0.5 MHz, acoustic pressure: 2–4 MPa, and pulse repetition frequency: 100 Hz).

## Highlights

• Increasing the volumetric concentration of PFP led to an increase in the size of lipid-coated PFP NDs.
• Increasing sonication intensity and time led to a decrease in ND size.
• The ND coating layer can be labelled with lipophilic fluorophores without affecting the overall ND size.
• NDs were stable at 4 °C for one week, and at 37 °C for 110 min.
• Ultrasound (0.5 MHz, 2–4 MPa) caused vaporisation of NDs within a tissue-mimicking phantom.

## 1 Introduction

Acoustically responsive microbubbles (MBs) have been extensively studied for both diagnostic and therapeutic applications over the last 40 years [1]. MBs are widely used in the clinic as contrast agents in ultrasound imaging (also known as ultrasound contrast agents, or UCAs) and, more recently, they have been investigated as therapeutic agents for the treatment of tumours, bacterial infections, neurological disorders, blood clots, and bone fractures [2]. Notably, the gas core of a MB is stabilised by an outer shell, which can also be employed as a scaffold to carry biologically active compounds [3], [4]. Upon exposure to an ultrasound field, MBs undergo volumetric oscillations (a process known as cavitation) and impart motion to the surrounding liquid medium, known as cavitation microstreaming. It has been postulated that these physical responses of a MB result in enhanced penetration of bio-active compounds into a target biological tissue [5].

Considering the gaseous nature of the MB core and their typical size (e.g., diameter range: 1–10 µm, and modal diameter < 3 µm [4]), MBs have a very short circulation time and experience only very limited extravasation when administered *in vivo* [6], which in turn may hinder their ability to effectively deliver a therapeutic payload within a target tissue. To address this limitation, there has been increasing interest in the formulation of nanoscale ultrasound-responsive agents, and particularly liquid perfluorocarbon (PFC) nanodroplets (NDs) with a stabilising shell (usually consisting of phospholipids, proteins or polymers). NDs exist as liquid emulsions with diameters in the range of 100–500 nm [4]. Whilst MBs have a circulation time of minutes (half-life < 5 min [7]), NDs have a half-life in the order of 2–6 h [6], [8], [9], [10], [11]. They also exhibit greater stability against pressure and mechanical stress imparted by the blood flow when compared to MBs [6], [12].

Once located at the target site, the liquid core of a ND can be induced to undergo liquid-to-gas phase transition upon exposure to ultrasound. This process is known as acoustic droplet vaporisation (ADV) and leads to the formation of gas-filled MBs. Nanodroplets are approximately five times smaller in diameter than the microbubbles they generate [6], [13], and the latter can be stimulated to promote drug delivery upon extracorporeal ultrasound stimulation [7]. Although the use of PFC droplets for therapy has gained significant interest over the last two decades, there are still open questions that may have hindered their clinical translation [9]. The size of PFC NDs is an important factor affecting their performance *in vivo*; notably, it is critical to find a trade-off between a ND that is small enough to extravasate and large enough to vaporise into a MB at relatively moderate acoustic pressures [3]. Previous studies have demonstrated extravasation of polymersomes and liposomes with a diameter < 200 nm [18], [19] and of paclitaxel loaded PFP PEG-PDLA NDs with an average size of 250–300 nm [20]. For this reason, a ND diameter in the range 200–300 nm is herein considered appropriate for achieving extravasation and subsequent therapeutic effects.

The exposure conditions required to achieve ND vaporisation can be varied by changing the composition of the ND core and/or size. The core is typically composed of a superheated liquid-state PFC, with PFCs of different molecular weight exhibiting different acoustic behaviours [8]. Most studies have reported that the smaller the ND, the higher the acoustic energy required to achieve phase transition [14]. The ND size distribution also influences their response to ultrasound stimulation [15] and their interaction with biological target cells [8]. For instance, if the size distribution is narrow, a large proportion of NDs in a suspension is likely to undergo vaporisation when exposed to a given set of ultrasound exposure conditions. Conversely, if the sample has a broad size distribution, only a limited proportion of NDs may vaporise [8], [16], [17].

NDs are normally produced as emulsions by mechanical agitation of a liquid PFC in the presence of an aqueous surfactant solution by using high-intensity low-frequency ultrasound (in the frequency range 20–25 kHz) [18] – a process known as sonication. It is a stochastic process causing the progressive break-up of the emulsion into smaller droplets, resulting in a highly concentrated suspension of surfactant-coated NDs [19]. PFC-loaded nanoparticles can also be produced *via* solvent evaporation from water-in-oil-in-water double emulsions (produced by sonication); this approach has been previously employed to produce poly-lactic-co-glycolic acid (PLGA) nanoparticles loaded with perfluorohexane (PFH) and gold nanoparticles [18]. Since sonication is the most convenient and commonly used manufacturing technique for PFC NDs within research settings (and is also employed industrially in the production of different classes of nano- and micro-particulate agents), it is worthwhile investigating whether production parameters can be optimised to tune ND size and size distribution to meet application-specific requirements; there has been some preliminary work in this area suggesting that this is possible. For instance, an investigation of the relationship between ND size and manufacturing parameters was carried out by Bilati *et al.*, who employed a two-step sonication technique to manufacture water-in-oil (first step) and water-in-oil-in-water (second step) emulsions, which subsequently formed PLGA nanocapsules through evaporation of the organic solvent. They showed that increasing sonication time and intensity led to a reduction of the emulsion’s mean diameter [20]. We aim to extend this work and apply it to a lipid-based PFC formulation.

The first part of this study investigates the effect upon ND size distribution of: (i) production parameters, specifically PFP concentration (5–15% v/v in PBS), sonication power (24–72 W) and sonication duration (20–60 s), and (ii) post-processing parameters, specifically temperature and fluorescent labelling with the lipophilic fluorophore DiI, which are relevant to experiments *in vitro* and *in vivo*. The second part of the study investigates phase transition of the manufactured NDs to establish the impact of nanodroplets characteristics upon acoustic droplet vaporisation.

## 2 Materials and methods

### 2.1 Production parameters

All NDs were produced by sonication, which is a method commonly used to manufacture nanoscale emulsions [8], [19]. The ND shell was composed of a phospholipid and an emulsifier, whilst the core was made of perfluoro-n-pentane (PFP, Strem, Newburyport, Massachusetts, USA), which is a PFC with a bulk phase transition temperature of 29 °C. Supplementary Figure D.1 illustrates the process of ND production. 1,2-distearoyl-*sn*-glycero-3-phosphocholine (DSPC, Avanti Polar Lipids, Alabaster, Alabama, USA) and polyoxyethylene(40)stearate (PEG40s, Sigma-Aldrich, Saint Louis, Missouri, USA) were dissolved in chloroform (Sigma Aldrich, UK) at 31.64 mM and 4.88 mM respectively, corresponding to a DSPC:PEG40s molar ratio of 9:1, which is commonly used in the production of lipid-shelled MBs [21]. Chloroform was left to evaporate overnight in a fume hood, and the resultant dry lipid film was subsequently hydrated in phosphate-buffered saline (PBS, Sigma-Aldrich, Saint Louis, Missouri, USA) to a final concentration of 3.94 mM and 0.44 mM for DSPC and PEG40s, respectively. PBS is a buffer solution (pH = 7.4) commonly used in biological research, as it is non-toxic to cells and its osmolarity and ionic concentrations are comparable to those of body fluids. Specifically, it is a water-based solution containing sodium chloride, sodium phosphate, and (in some formulations) potassium chloride and potassium phosphate. The mixture was heated to 95 °C for 30 min, while mixing with a magnetic stirrer (at 700 rpm); and was then sonicated a first time for 15 s, with the tip of the sonicator completely immersed in the liquid phase in order to homogeneously disperse the lipids. The mixture was then cooled to 4 °C for 30 min, prior to the addition of PFP to a final concentration between 5 and 15% (v/v in PBS). Upon addition of PFP, the vial was then agitated for 2 s using a vortex mixer (SciQuip Ltd, Newtown, UK) to generate a micro-emulsion.

A nano-emulsion was subsequently generated *via* sonication of the micro-emulsion. A 1 mL aliquot of the micro-emulsion was placed in a 1.5 mL Eppendorf tube (Starlab, Milton Keynes, UK) and sonicated at 22.5 kHz for a total active time (i.e., the total length of time during which the sonicator was in the ‘ON’ mode) between 20 and 60 s at a power of 24–72 W, using a 3.22 mm diameter tip sonicator (Fisher Scientific, Hampton, New Hampshire, USA), with the tip completely immersed in the liquid. This second sonication was performed either in a continuous or pulsed mode (at duty cycle of 15% or 30%), but maintaining a constant active time. The ND suspension was kept on ice to ensure that the temperature did not rise above the phase transition temperature of PFP.

### 2.2 Parameters and conditions relevant for experiments *in vitro* and *in vivo*

The effect of the incorporation of a fluorophore in the ND preparation was investigated by addition of the lipophilic dye 1,1′-dioctadecyl-3,3,3′,3′-tetramethylindocarbocyanine perchlorate (DiI, Life Technologies, Carlsbad, California, USA) at final concentrations of 2.14, 10.71 and 21.42 µM, corresponding to molar ratios DSPC:PEG40S:DiI of 89:10.1:0.025, 90:10.1:0.12 and 90:10.1:0.25. Labelling of NDs with fluorescent dyes facilitates multimodal imaging to better visualise and track NDs in living organisms. Notably, lipophilic dyes such as DiI, DiO and DiD are commonly used to label lipid-coated MBs and NDs [7], [22], [23], [24]. Detection of labelled NDs using a fluorescence microscope and their interaction with biological cells was also demonstrated, by following the method presented in Appendix C.

To investigate the stability of NDs, they were stored at both 4 °C (a typical storage temperature) in a fridge, and 37 °C (physiological temperature) in an incubator (NuAire, Plymouth, Minnesota, USA). Samples were imaged every 10 min with an optical microscope at a magnification of 50x (Olympus IX71, Olympus, Shinjuku, Tokyo, Japan) to perform a qualitative evaluation of the quality of the sample, while ND size was quantified by dynamic light scattering (DLS, Zetasizer Nano, Malvern Panalytical Ltd, Malvern, UK) within a 1 mL cuvette (Fisherbrand FB55147, Fisher Scientific, UK) at a 1:2 v/v dilution in PBS. ND diameter was measured soon after production, and every 10 min for >2 h. Three independent samples were measured at each time point tested.

### 2.3 Transition from NDs to MBs

The acoustic response of NDs depends on different factors, including the physical conditions of the surrounding medium. The acoustic response of PFP NDs was therefore measured in a tissue-mimicking flow phantom with an embedded 1 mm diameter channel, through which the ND suspension was conveyed. The experimental setup is described in earlier studies [25], and is shown in Fig. 4.A. Briefly, a focused ultrasound (FUS) transducer (H103, Sonic Concepts, Bothell, WA, USA) of fundamental frequency 0.5 MHz was used to stimulate the NDs. The aperture and the geometric focus of the transducer were 64 mm and 60 mm, respectively. A 7.5 MHz spherically focused passive cavitation detector (PCD, V320 Panametrics, Olympus, Waltham, USA), of element diameter 12.5 mm and focal length 75 mm, was used to record any acoustic emissions. The tissue-mimicking phantom was made from a degassed biocompatible hydrogel composed of 2% (w/v) low melting point ultrapure agarose gel (Invitrogen, Carlsbad, CA, USA) in deionised water. The FUS transducer and PCD were coaxially and confocally aligned *via* a central circular opening in the FUS transducer, which focused the middle of the agarose channel in the tissue-mimicking phantom. Both FUS transducer and PCD were fully controlled using a custom-made software (LabVIEW, National Instruments, Austin, TX, USA). The degassed suspension of NDs was injected through the flow phantom at 0.4 mL/min using a syringe pump (Pump 11 Elite, Harvard Apparatus, Massachusetts, USA) and exposed to the ultrasound field. The experiment was performed at a temperature of 20 °C. The frequency spectra of the emissions recorded by the PCD were used to determine the acoustic pressure threshold required to induce ND vaporisation and the acoustic response of the resulting microbubbles. Two thousand-cycles FUS excitation pulses of increasing pressure (2–4 MPa; corresponding to a mechanical index, MI, of 2.8-5.7) were transmitted whilst a continuous flow of NDs passed through the phantom, allowing for complete replenishment of the exposed sample between each pulse. The pulse repetition frequency (PRF) of ultrasound was set to 100 Hz. A Philips iU-22 ultrasound scanner (Philips Ultrasound, Bothell, WA, USA) was used to provide real-time B-mode imaging to detect ND vaporisation during the experiment. The imaging also allowed monitoring of the physical integrity of the channel, detecting possible leaks, and ensuring that the flow did not contain exogenous air bubbles. Harmonic imaging was used at a low mechanical index (MI = 0.1) in order to avoid any interference with both the excitation pulses and the signal generated by NDs.

For the above tests, NDs were produced following the protocol previously presented, with the following parameters: 10% PFP (v/v in PBS), second sonication performed for 60 s at 72 W and 30% duty cycle. NDs were centrifuged twice at 10,000 g for 5 min with washing in between, during which the supernatant was aspirated and NDs were re-suspended in fresh PBS. After centrifugation and washing, the ND suspension had a mean diameter of approximately 400 nm. To study the effect of ND size on the acoustic exposure parameters needed to induce ND vaporisation, the sample was passed through a 450 nm filter (Whatman Uniflo Syringe Filters, GE Healthcare Life Sciences, Little Chalfont, UK) to remove larger particles and obtain a suspension with a mean diameter of approximately 200 nm.

### 2.4 Data plotting and statistical analysis

Results relating to the diameter and size distribution of the batches analysed are reported in the form of boxplots to indicate the distribution of the data thanks to the subdivision into quartiles. Each box thus illustrates the following values: minimum (bottom whisker), 1st quartile (bottom line of the box), median (intermediate line of the box), 3rd quartile (top line of the box), and maximum (top whisker). Statistical analysis of the experimental data was also carried out. One-way analysis of variance (ANOVA) was the chosen statistical test and was performed using SPSS (IBM Analytics, Armonk, New York, USA). ANOVA was used to determine whether there were any statistically significant differences between the means of independent groups. However, the ANOVA test does not define which are the specific groups that are statistically different and, to do so, a post hoc test was carried out. The post hoc test performed in this study was the Tukey's honestly significant difference (HSD).

## 3 Results & discussion

### 3.1 The effect of production parameters on ND size and size distribution

#### 3.1.1 Effect of varying the volumetric concentration of PFP

First, we tested the effects of varying the volumetric concentration of PFP on ND size. We hypothesised that increasing the concentration of PFP would lead to an increase in the diameter of the emulsion droplets formed by sonication, due to the corresponding decrease in the concentration of phospholipid surfactant relative to the amount of PFP. NDs were produced with a second sonication having a total active time of 20 s and a power of 72 W.

**Fig. 1:**
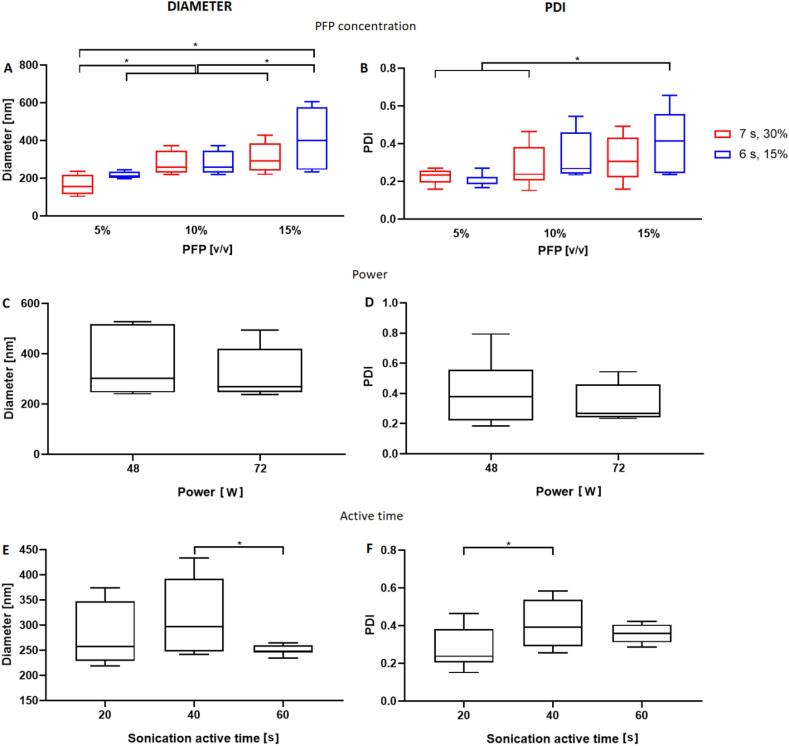
Effects of production parameters on ND size and size distribution. A,B) Effect of the quantity of PFP and the pulse length of the second sonication on ND size and size distribution. In these experiments, NDs were produced by applying a pulsed sonication of 20 s at 72 W, with either a pulse of 7 s and 30% duty cycle (red) or a pulse of 6 s and 15% duty cycle (blue). On the left, boxplots show the ND diameter distribution (just after production). NDs produced with 5% (v/v) PFP and a longer pulse were statistically different from the other groups tested (*p < 0.05). On the right, boxplots show the PDI of NDs (just after production). C,D) Effect of second sonication power on ND size and size distribution. Sonication was performed for a total active time of 20 s, with a pulse of 7 s and 30% duty cycle. On the left, boxplots show the distribution of ND diameter after production, for NDs obtained with sonication at power of 48 W and 72 W. An increase in the sonication power resulted in a decrease in ND mean size and size distribution. On the right, boxplots show the PDI of ND after production, for NDs obtained with sonication at power of 48 W and 72 W. Increasing the sonication power led to a reduced polydispersity index. E,F) Effect of second sonication active time on ND size and size distribution. Sonication was performed in a pulsed mode with a pulse of 7 s and 30% duty cycle. On the left, boxplots show the diameter distribution of NDs produced with different durations of the second sonication. There was a statistical difference between NDs produced with a sonication of 40 s and 60 s (*p < 0.05). On the right, PDI of NDs produced with different durations of the second sonication. There was a statistical difference between NDs produced with a sonication of 20 s and 40 s (p < 0.05). Three different samples were tested for each experimental condition reported in the graphs (N = 3). (For interpretation of the references to colour in this figure legend, the reader is referred to the web version of this article.)

Supporting this hypothesis, we found that increasing the concentration of PFP from 5 to 15% (v/v) led to an increase in the mean ND diameter from 162.9 ± 49.4 nm to 308.7 ± 76 nm (Fig. 1.A,B and supplementary Table B.1). The size dispersity of NDs also increased with increasing the concentration of PFP; i.e. the polydispersity index (PDI, a measure of the broadness of the size distribution) increased from 0.23 to 0.32 (p < 0.05) when increasing the quantity of PFP from 5 to 15% (v/v). Notably, as PFP is immiscible in the saline dispersion of DSPC and PEG40s, it initially formed a separate layer at the bottom of the vial. The vial was thus subsequently stirred (for 2 s) to break this layer into droplets. During the second sonication, these droplets were broken down into nano-sized droplets, as previously described by Anton *et al*. [21]. Therefore, it is likely that a greater amount of PFP results in larger precursor droplets that - in turn - generate larger NDs upon sonication, with a broader size distribution. Moreover, in this experiment, greater amounts of PFP were associated with a decrease in the relative concentration of phospholipid surfactant, ultimately resulting in larger NDs to optimise the surface-area-to-volume ratio with respect to the available amount of surfactant. This behaviour has already been reported in previous studies; for instance, Walker *et al.* showed that as the surfactant-to-oil ratio increased in fish oil nanoemulsions, the mean particle diameter decreased [30]. Zirak *et al.* also found that greater surfactant concentrations decreased the surface tension and stabilised newly developed surfaces during the production of lipid nano-carriers [31]. Therefore, a reduction in the phospholipid concentration relative to the amount of PFP may likely affect the phospholipid packing density and the interfacial tension, which would in turn likely impact upon ND stability [26].

Thus, adjusting the quantity of PFP in solution offers a means of controlling the final average ND diameter of the batch. Since our identified ND target diameter is in the range 200–300 nm, 10% PFP (v/v in PBS) is the preferred concentration among those investigated in this study.

#### 3.1.2 Effect of varying the second sonication pulse length

We tested the effects of varying the second sonication pulse length on ND size, hypothesizing that shorter sonication pulses would lead to larger NDs. Indeed, bulk PFP has a liquid-to-gas phase transition temperature of 29 °C. Thus, an increase in fluid temperature during the second sonication process may cause undesired evaporation of PFP, which may in turn affect the properties of the obtained ND suspension. By shortening the sonication pulse, the increase in temperature could be reduced, thus reducing the extent of PFP evaporation and resulting in a greater amount of PFP available in the medium (hence larger NDs).

A reduced second sonication power and duration (pulse length of 6 s and 15% duty cycle compared to 7 s with 30% duty cycle), resulted in larger NDs with greater size dispersity. However, there was no significant change (p > 0.05) in the final ND size and size dispersity by increasing the pulse duration and duty cycle (Fig. 1.A,B, supplementary Table B.1 and Table B.2, for each amount of PFP tested). The difference between the two pulsation regimes was thus not sufficient to cause a significant difference in size between the produced NDs. Although there was no statistical difference between groups, NDs produced with a longer pulse were slightly smaller, by 40–50 nm, and had a reduced size dispersity relative to the NDs produced using a shorter pulse (Fig. 1.A,B, supplementary Table B.1 and Table B.2). This might be due to two reasons: the more persistent mechanical effects induced by sonication on one side, and increased PFP evaporation on the other (hence, less PFP available when longer pulses are used).

A narrower size distribution may be preferable for acoustic activation as it leads to a more homogeneous and predictable acoustic response [30], hence longer sonication times (among the ones investigated) may be advantageous.

#### 3.1.3 Effect of varying the power of the second sonication step

We next hypothesised that increasing the intensity of the second sonication would lead to a corresponding decrease in ND size and size dispersity, due to a more vigorous mechanical breakdown of the NDs. NDs were produced using 10% PFP (v/v in PBS), and through a second sonication of 6 s with 15% duty cycle and total active time of 20 s. These sonication settings led to greater variability in the average ND size in previous experiments, and were thus selected as the preferred experimental conditions to assess whether increasing the sonication power would reduce NDs’ dimensional variability. Confirming this, both ND size and size dispersity reduced from 354.6 ± 127.2 nm to 315. ± 100.5 nm when sonication power was increased from 48 to 72 W (Fig. 1.C,D and supplementary Table B.3). A lower power of 24 W was also employed; however, the corresponding sample size distribution was too broad to be accurately quantified by DLS.

The decrease in the mean size and size dispersity of NDs produced at the higher sonication power could be due to the greater mechanical effects associated with a higher ultrasound intensity, namely shear stress and pressure generated by cavitation and streaming, that may cause further breakdown of newly formed NDs. This is further corroborated by the fact that a sonication intensity of 24 W led to a sample of insufficient quality (i.e., with excessive size dispersity) to be analysed. These results are in line with previous observations by Bilati *et al*. [20], where higher sonication intensities led to smaller polymer-shelled NDs. This also shows that the sonication intensity has similar effects on the size of both lipid-shelled and polymer-shelled NDs.

#### 3.1.4 Effect of varying the length of the second sonication step

The active time of the second sonication step was varied between 20 and 60 s. The underlying hypothesis was that an increase in the sonication length would lead to smaller NDs with a lower size dispersity, due to the longer mechanical action of sonication on the batch. NDs were produced using 10% PFP (v/v in PBS), and through a second sonication of 7 s with 30% duty cycle and a power of 72 W. When sonication was increased from 20 to 40 s (maintaining the pulse duration, power and PFP volume constant) there was no significant change in the size of NDs, while the PDI increased marginally from 0.27 to 0.41 (p < 0.05); however, this trend was reversed when the sonication time was increased by a further 20 s, with a significant decrease in ND diameter. It was also observed that the ND size was significantly more consistent across repeats when using a longer second pulse, with standard deviations decreasing for both diameter (from 75.28 nm to 9.67 nm) and PDI (from 0.12 to 0.04) (Fig. 1.E,F, and supplementary Table B.4).

These results obtained using lipid-shelled NDs are in line with previous findings by Bilati *et al*. [20], where a longer sonication led to the formation of smaller polymer-shelled NDs. The more persistent mechanical effects of a longer sonication could allow overcoming the variability between multiple repeats, suggesting that some of the stochastic characteristics of the process are averaged out over longer time scales. Based on the results shown in Fig. 1.A,B, another possible reason for obtaining smaller lipid-shelled NDs at longer active sonication time could be due to the evaporation of PFP.

For the 20 s sonication, the mean diameter had a standard deviation of 60.7 nm, while the mean diameter of NDs obtained with a 60 s sonication had a smaller standard deviation of 9.7 nm. Therefore, among the different conditions investigated, a sonication of 60 s was regarded as the preferred procedure to obtain NDs of therapeutic utility.

### 3.2 The effect of post-processing parameters on ND size and size distribution

#### 3.2.1 Effect of labelling the nanodroplet shell with a lipid-analogue dye

**Fig. 2:**
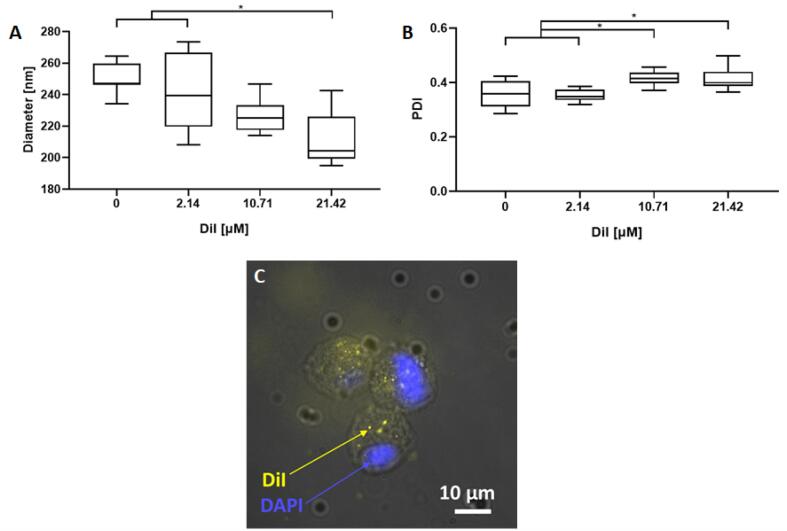
Effect of the quantity of DiI on ND size and size distribution. NDs were sonicated for a total active time of 60 s at 72 W, with a pulse of 7 s and 30% duty cycle. A) The boxplots show the diameter of NDs produced with different amounts of DiI. Increasing the quantity of DiI led to a small decrease in the size of NDs from 240.5 ± 23.7 to 211.8 ± 16.7 nm (*p < 0.05) B) Boxplots showing the polydispersity index (PDI) of NDs produced with different amounts of DiI. Three different samples were tested for each case (N = 3). C) The microscope image (40x oil immersion) shows three MG63 cells, where the blue circle is the cell nucleus stained with DAPI dye, while the yellow spots are NDs stained with DiI. (For interpretation of the references to colour in this figure legend, the reader is referred to the web version of this article.)

In this study, NDs were labelled with DiI at different molar ratios, to assess whether the lipid-analogue dye could have an impact on their size. Three different molar ratios were used, DSPC:PEG40s:DiI of 89:10.1:0.025 (2.14 µM), 90:10.1:0.12 (10.71 µM), and 90:10.1:0.25 (21.42 µM). NDs were produced using 10% PFP (v/v in PBS), through a second sonication of 7 s with 30% duty cycle, power of 72 W, and total active time of 60 s. Increasing the quantity of DiI led to a small decrease in the size of NDs from 240.5 ± 23.7 to 211.8 ± 16.7 nm (p < 0.05), as shown in Fig. 2.A,B and supplementary Table B.6. Talu *et al*. [17] showed that a molar ratio of 0.9:0.101:0.000196 is sufficient to permit fluorescence microscopy imaging of NDs, thus suggesting that the lowest amount of DiI employed in this study would still be sufficient for ND imaging. Confirming this, DiI-labelled NDs were observed to induce intracellular fluorescence in MG63 cells, as shown in Fig. 2.C.

In conclusion, DiI in the range 2.14–21.42 µM can be used in conjunction with lipid-shelled NDs to label them without affecting their size and stability upon manipulation, and to study their interaction with biological cells.

#### 3.2.2 Effect of storage temperature

**Fig. 3:**
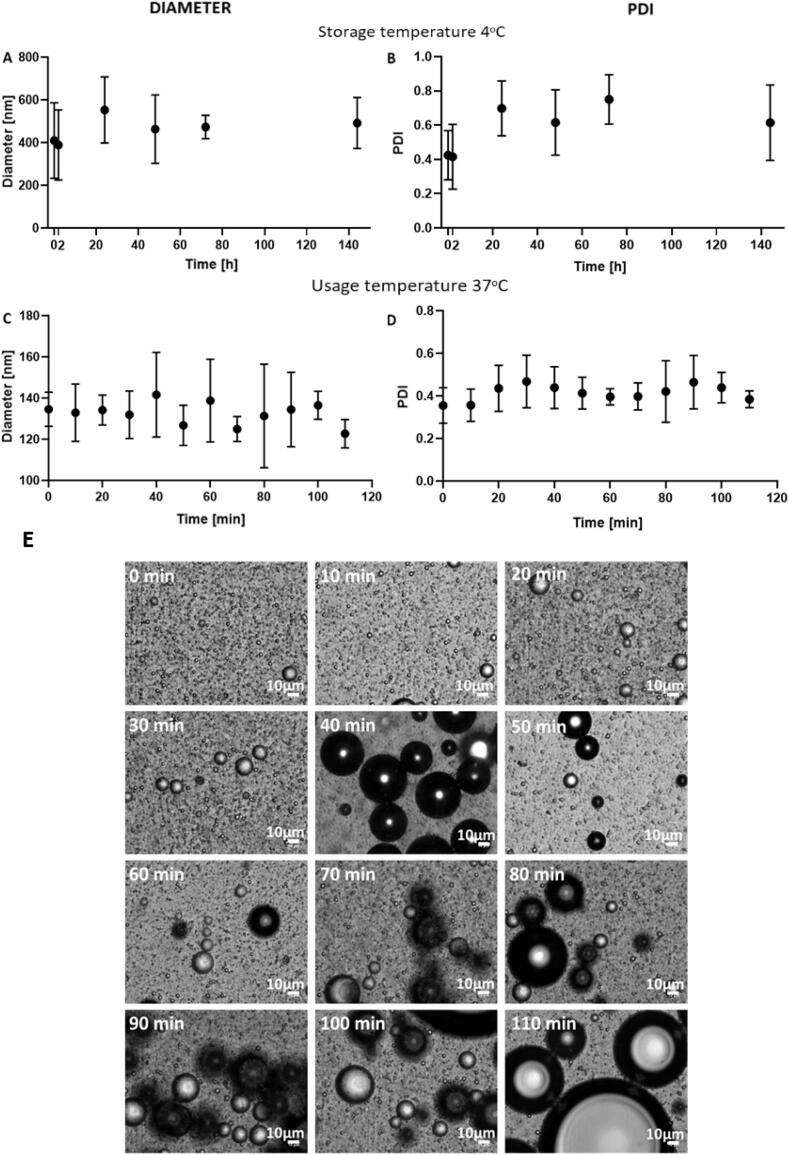
ND stability over time. A,B) ND stability over 1 week at 4 °C. NDs were produced by sonication for a total active time of 60 s at 72 W, with a pulse of 7 s and 30% duty cycle. On the left, the graph shows the diameter of NDs over time. There was no statistical difference (p > 0.05) between diameters measured at different time points. On the right, boxplots showing the polydispersity index of NDs, at the different time points tested. Values obtained after production and after two hours were smaller (p < 0.05) than the other values. C,D) ND stability over 110 min at 37 °C. NDs were sonicated for a total active time of 60 s at 72 W, with a pulse of 7 s and 30% duty cycle. On the left, the graph shows the mean ND diameter and standard deviation over time. There was no statistical difference (p > 0.05) between the different values obtained. On the right, boxplots show the ND PDI and its standard deviation. No statistical difference was found (p > 0.05) between these values. Three different samples were tested for each case (N = 3). E) Bright field microscope images (50x magnification) taken from a sample of NDs stored at 37 °C. Pictures were taken every 10 min, up to 110 min. Some small MBs were present just after production; from 40 min onwards, larger MBs were detected.

NDs produced with 10% PFP (v/v in PBS), a second active sonication of 20 s at 72 W and with a pulse length of 7 s and 30% duty cycle, were stored at 4 °C and their size was measured at different time points to study their storage stability, which is an important factor for potential clinical translation. There was no significant change in the diameter of NDs with time (p > 0.05). PDI values obtained after production and after 2 h were smaller than the ones obtained at the later time points tested (p < 0.05, Fig. 3.A,B, and supplementary Table B.5).

The size dispersity of NDs increased over time (PDI = 0.4 after production and after two hours, PDI > 0.6 at greater time points). According to the results obtained in this study, if NDs are used up to two hours after production, their polydispersity index is maintained almost constant, allowing safe administration. This suggests that NDs should be administered soon after production, potentially within the first two hours, in order to ensure that the preparation preserves a relatively homogeneous size. In the literature, there is no available quantitative study using the same ND formulation to directly compare and validate these results with; however, Zhang *et al*. studied the stability of PEGylated PLGA-based NDs at 4 °C for 5 days by using B-mode imaging. They found that NDs were stable for up to two days from production; from the third day, potential formation of MBs was observed [27]. Phase transition of PFP may also be the reason for the observed change in ND size distribution in the present study, but microscopic observations would be required to confirm this hypothesis.

#### 3.2.3 Effect of exposure to physiological temperature

We next examined the short-term stability of NDs at a physiological temperature (37 °C). NDs were produced using a second sonication step with a total active time of 60 s at 72 W, with a pulse length of 7 s and 30% duty cycle, and were stored at 37 °C for two hours in PBS.

The ND diameter did not change over time, as there was no statistically significant difference between the diameter of the samples analysed (p > 0.05), as shown in Fig. 3.C,D and supplementary Table B.7. There is no available study for comparison using the same chemical composition and investigating ND stability at 37 °C. However, Lee *et al*. showed that the average size of magnetic particle-loaded dodecyl(trimethyl)ammonium bromide (DTAB) PFP NDs increased over time at 37 °C, and their concentration halved after 6 h from production, while the concentration of Albumin–PEG hybrid nanoparticles encapsulating superparamagnetic iron oxide and PFP reduced by just 3.5% in the first 6 h, denoting a significant dependence of stability on the ND shell composition [28].

Microscope images of the samples were also acquired at each time point analysed, and showed the likely presence of some MBs just after production, as illustrated in Fig. 3.E, which could not be detected by DLS. After production, MBs present in the sample had a mean diameter of 2.48 ± 1.06 µm, while the largest MB detected at this time point had a diameter of 10.5 µm. This demonstrates that the particle size in the sample is still below the critical value for *in-vivo* administration of 10 µm (95% of the MBs in batches used in the clinic have a size smaller than 10 µm [29]). It is thought that the larger MBs (diameter > 30 µm) appearing from 40 min onwards are the result of the coalescence of small MBs (diameter < 10 µm) and nanobubbles present in the sample from the very beginning, and not due to vaporisation - since the expected transition temperature for these NDs is approximately 70 °C [30].

### 3.3 Phase transition from NDs to MBs

**Fig. 4:**
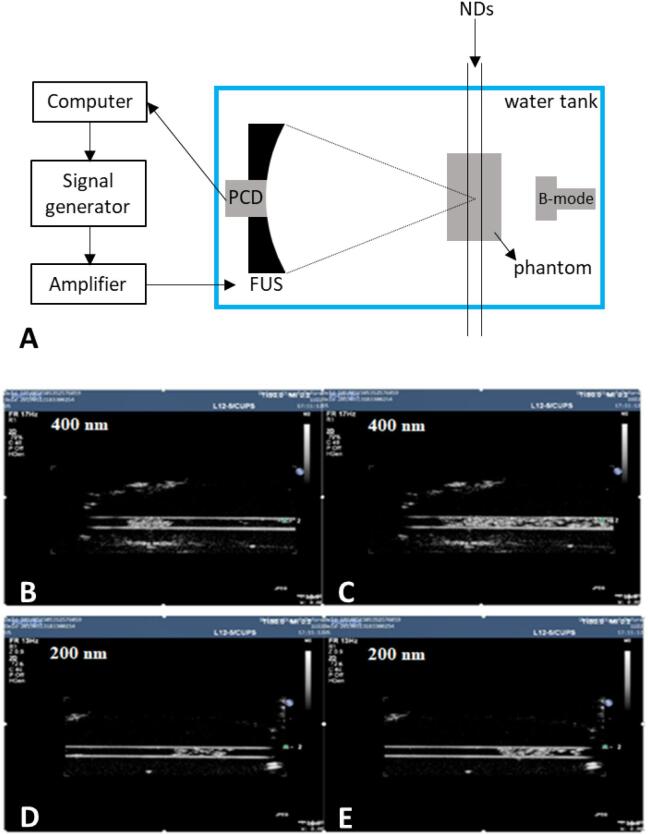
Setup and real time B-mode images of the channel containing PFP NDs. A) Schematic diagram of the experimental setup employed to investigate the acoustic response of NDs. The suspension of NDs was conveyed through a flow phantom at 0.4 mL/min and exposed to 0.5 MHz ultrasound emitted by a FUS transducer. A coaxially and confocally aligned PCD was used to record ND emissions. B) B-mode image showing the phase transition of PFP NDs with an initial mean diameter of 400 nm. C) B-mode image of MBs obtained from the phase transition of PFP NDs with a mean diameter of 400 nm. D) B-mode image showing the phase transition of PFP NDs with an initial mean diameter of 200 nm. E) B-mode image of MBs obtained from the phase transition of PFP NDs with a mean diameter of 200 nm.

To verify and visualise liquid-to-gas phase transition of PFP NDs in a tissue-mimicking flow phantom, real-time B-mode imaging was used. Both 400 nm and 200 nm NDs were tested, and the acoustic pressure at which phase transition occurred was recorded. Fig. 4.B and Fig. 4.D show the moment at which phase transition from NDs was first detected and Fig. 4.C and Fig. 4.E show the channel filled with the MBs generated by phase transition.

Phase transition of the larger NDs (i.e. with a mean diameter of 400 nm) was first detected within the channel at an acoustic pressure of 3 MPa (MI: 4.2). As for the sample containing the 200 nm NDs, phase transition was firstly observed when an acoustic pressure of 3.87 MPa was applied (MI: 5.5). The PFC chosen for this work was PFP since it has a phase transition temperature of 29 °C, meaning that it is in its liquid phase at ambient temperature. Once in a droplet configuration, its phase transition temperature increases due to the surface tension between the PFC and the surrounding medium. The boiling point of a ND is related to the acoustic energy required to vaporise them by the Clausius-Clapeyron equation; the smaller the droplet, the higher its phase transition temperature, hence the greater the peak negative acoustic pressure required to vaporise it. This is consistent with the observations reported in this study [4]. It should be noted that the experiment was performed at 20 °C; however, NDs will experience a greater temperature *in vivo* (≈37 ^o^C), which is likely to decrease the required acoustic vaporisation pressure.

Overall, these experimental observations demonstrate that the protocol and chemical composition used to formulate NDs in this study were suitable to achieve ND vaporisation at conditions relevant for *in vivo* usage, namely flow dynamic conditions and ultrasound settings that are within the range of therapeutic applications. It should be noted that the ultrasound field properties employed in this study differ from those typically generated by standard clinical scanners; therefore, *in vivo* assessment of ND performance would require custom-designed ultrasound systems tailored to a desired therapeutic application. Moreover, the acoustic pressure levels employed in this study are likely to induce cavitation effects *in vivo*, which would also need to be assessed in relation to a specific therapeutic application. Whilst in the present study we aimed to determine whether the produced lipid-shelled PFC nanodroplets could undergo acoustic vaporization, future studies will evaluate more comprehensively the effect of ultrasound parameters on NDs’ acoustic vaporisation.

## 4 Conclusion

The experiments reported in this study investigated the effect of processing and handling parameters on the size characteristics of lipid-shelled PFC nanodroplets, with the aim of developing a protocol to produce NDs with a clinically acceptable size and size distribution.

It was found that increasing the volumetric concentration of PFP caused an increase in ND size, while increasing the power of sonication and the total sonication time led to decreases in both size and size dispersity. NDs were also stained with a lipophilic dye (DiI, with a final concentration in the range 2.14–21.42 µM) and the effect of labelling on ND size was found to be negligible.

To obtain lipid-shelled NDs in the therapeutically favourable size range of 200–300 nm, it is therefore advisable to add 10% of PFP (v/v in PBS) to the lipid solution prior to the second sonication. The parameters of the second sonication are fundamental to achieve the desired ND characteristics, and an optimal protocol was identified to be: pulsed second sonication of 7 s and 30% duty cycle, total active sonication time of 60 s, and sonication intensity of 72 W. NDs produced with this protocol were stable at both storage temperature (4 °C) for one week and at body temperature (37 °C) for up to 110 min. Moreover, NDs were observed to undergo phase transition to gas microbubbles upon US stimulation in a tissue-mimicking phantom (at ultrasound frequency of 0.5 MHz, acoustic pressure in the range 3–4 MPa, pulse repetition frequency of 100 Hz, and temperature of 20 °C).

The findings from this study could inform researchers aiming to produce batches of PFC NDs with a specific size range, in order to obtain reliable extravasation *in vivo* and subsequent vaporisation at the therapeutic target site. Future studies will further investigate the stability and acoustic behaviour of PFP nanodroplets, upon exposure to a range of different physico-chemical conditions and therapeutically relevant ultrasound parameters.

## 5 Funding sources

This work was supported by the Institute for Life Sciences (University of Southampton), the Medical Research Council (MRC) NPIF 2017 fund (aligned to MRC DTP in Translational Immunology), and the EPSRC grant “Bubbles to Bond Broken Bones: targeted drug delivery for fracture repair” (EP/R013624/1 and EP/R013594/1). Data from this study will be made available through an online repository.

## Declaration of Competing Interest

The authors declare that they have no known competing financial interests or personal relationships that could have appeared to influence the work reported in this paper.
