# Supplementary material for: Tailoring the size of ultrasound responsive lipid-shelled nanodroplets by varying production parameters and environmental conditions
Source: Ultrason Sonochem. 2021 Feb 3;73:105482. doi: 10.1016/j.ultsonch.2021.105482 (PMC7901031; doi:10.1016/j.ultsonch.2021.105482)
Supplement: Supplementary data 1 [file mmc1.docx]

# Supplementary information for:

**Tailoring the size of ultrasound responsive lipid-shelled nanodroplets by varying production parameters and environmental conditions.**

Sara Ferri^1,2,4^, Qiang Wu^3^, Antonio De Grazia^1^, Anastasia Polydorou^1,2^, Jonathan P. May^1,2^, Eleanor Stride^3^, Nicholas D. Evans^1,2,4^, Dario Carugo^5*^

^1^Faculty of Engineering and Physical Sciences, Department of Mechanical Engineering, University of Southampton, UK

^2^Centre for Human Development, Stem Cells and Regeneration, Bioengineering Sciences, Faculty of Medicine, University of Southampton, UK

^3^Department of Engineering Science, University of Oxford, UK

^4^Institute for Life Sciences (IfLS), University of Southampton, UK

^5^Department of Pharmaceutics, School of Pharmacy, University College London (UCL), UK

*Corresponding Author: Dario Carugo

Present/Permanent Address: Department of Pharmaceutics, School of Pharmacy, University College London (UCL) (d.carugo@ucl.ac.uk)

1. **B-mode imaging of the ND suspension within a tissue-mimicking flow phantom**

Figure A.1 shows a real-time image of the channel containing a sample of NDs prior to US stimulation.


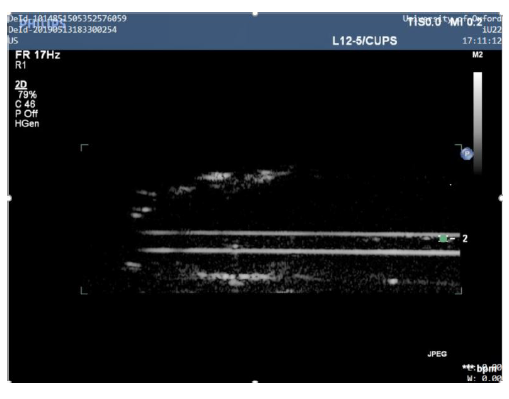


Figure A.1. Real-time B-mode image of the channel containing PFP NDs, prior to US stimulation.

1. **Numerical data corresponding to the experiments performed in this study**

Table B.1 Effect of varying the volumetric concentration of PFP. The table shows the mean diameter, polydispersity index and relative standard deviations of mean values for NDs (measured just after production), obtained using different quantities of PFP in the range 5-15% (v/v) and sonicated for a total active time of 20 s at 72 W, with a pulse of 7 s and 30% duty cycle.

| PFP  [% v/v] | Diameter  [nm] | PDI |
| --- | --- | --- |
| 5 | 162.9 ± 49.4 | 0.23 ± 0.03 |
| 10 | 277.5 ± 60.7 | 0.27 ± 0.11 |
| 15 | 308.2 ± 76 | 0.32 ± 0.11 |

Table B.2 Effect of varying the second sonication pulse length. The table shows the mean diameter, polydispersity index and relative standard deviations of mean values for NDs (measured just after production) obtained using different quantities of PFP in the range 5-15% (v/v), and sonicated for a total active time of 20 s at 72 W, with a pulse of 6 s and 15% duty cycle.

| PFP  [% v/v] | Diameter  [nm] | PDI |
| --- | --- | --- |
| 5 | 215.8 ± 16.8 | 0.20 ± 0.03 |
| 10 | 315 ± 100.5 | 0.33 ± 0.12 |
| 15 | 408.9 ± 171.2 | 0.42 ± 0.17 |

Table B.3 Effect of varying the intensity of the second sonication step. The table shows the mean diameter, polydispersity index and relative standard deviations of mean values for NDs (measured just after production) obtained using different sonication powers of 48 and 72 W. Sonication was performed for a total active time of 20 s, with a pulse of 6 s and 15% duty cycle.

| Intensity  [W] | Diameter  [nm] | PDI |
| --- | --- | --- |
| 48 | 354.6 ± 127.2 | 0.41 ± 0.20 |
| 72 | 315 ± 100.5 | 0.33 ± 0.12 |

Table B.4 Effect of varying the length of the second sonication. The table shows the mean diameter, polydispersity index and relative standard deviations of mean values for NDs produced with different sonication active times. Sonication was performed with a pulse of 7 s and 30% duty cycle.

| Time  [s] | Diameter  [nm] | PDI |
| --- | --- | --- |
| 20 | 277.5 ± 60.7 | 0.27 ± 0.11 |
| 40 | 314 ± 75.3 | 0.41 ± 0.12 |
| 60 | 249.7 ± 9.7 | 0.35 ± 0.04 |

Table B.5 NDs stability over time. The table shows the mean diameter (and corresponding standard deviation) of NDs at the different time points tested during storage at 4^o^C, and corresponding polydispersity index. NDs were sonicated for a total active time of 20 s at 72 W, with a pulse of 7 s and 30% duty cycle.

| Time  [h] | Diameter  [nm] | PDI |
| --- | --- | --- |
| 0 | 409.9 ± 176.7 | 0.42 ± 0.14 |
| 2 | 389 ± 164.4 | 0.41 ± 0.19 |
| 24 | 553.2 ± 155.2 | 0.70 ± 0.16 |
| 48 | 463.1 ± 160.2 | 0.61 ± 0.19 |
| 72 | 473.2 ± 55.2 | 0.75 ± 0.14 |
| 144 | 491.7 ± 119.3 | 0.61 ± 0.22 |

Table B.6 Effect of labelling the ND shell with a lipid-analogue dye. The table shows the mean diameter, polydispersity index and relative standard deviations of mean values for NDs produced with different quantities of DiI. The second sonication was performed for a total active time of 60 s at 72 W, with a pulse of 7 s and 30% duty cycle.

| DiI  [µM] | Diameter  [nm] | PDI |
| --- | --- | --- |
| 2.14 | 240.5 ± 23.7 | 0.35 ± 0.02 |
| 10.71 | 226.2 ± 10.4 | 0.44 ± 0.07 |
| 21.42 | 211.7 ± 16.7 | 0.41 ± 0.04 |

Table B.7 Effect of storage temperature. The table shows the mean diameter, polydispersity index and relative standard deviations of mean values for NDs stored at 37^o^C and analysed at different time points, up to 110 min. The second sonication was performed for a total active time of 60 s at 72 W, with a pulse of 7 s and 30% duty cycle.

| Time  [min] | Diameter  [nm] | PDI |
| --- | --- | --- |
| 0 | 134.6 ± 8.3 | 0.35 ± 0.08 |
| 10 | 132.9 ± 13.9 | 0.36 ± 0.08 |
| 20 | 134.1 ± 7.3 | 0.43 ± 0.11 |
| 30 | 131.9 ± 11.5 | 0.47 ± 0.12 |
| 40 | 175 ± 67 | 0.44 ± 0.10 |
| 50 | 126.8 ± 9.8 | 0.41 ± 0.07 |
| 60 | 138.7 ± 20.1 | 0.39 ± 0.04 |
| 70 | 125 ± 6.1 | 0.39 ± 0.06 |
| 80 | 131.3 ± 25.1 | 0.42 ± 0.14 |
| 90 | 134.4 ± 18.1 | 0.46 ± 0.12 |
| 100 | 136.5 ± 6.8 | 0.44 ± 0.07 |
| 110 | 122.7 ± 6.9 | 0.38 ± 0.04 |

1. **Visualising intracellular uptake of nanodroplets: experimental protocol**

A preliminary test was performed to assess whether the produced DiI-labelled NDs are likely to be taken up by cells and can be visualised using a fluorescence microscope. A flask with 400’000 human osteosarcoma MG63 cells was prepared and maintained in Dulbecco’s Modified Eagle’s medium (DMEM, Lonza, Basel, Switzerland) supplemented with 5% (v/v) foetal bovine serum (FBS, Gibco, UK), 100 U/mL penicillin, and 100 µg/mL streptomycin. Cells were then incubated at 37^o^C and 5% CO_2_ for 24 hours, to reach a confluence of around 1 million cells. After 24 hours, the medium was removed and discarded in Virkon, a multi-purpose disinfectant, and the flask was treated as follows: 9.5 mL of fresh medium was added along with 500 µL of ND solution, and cells were incubated for 40 min. After 4 min, the medium was removed, cells were washed twice with PBS and detached from the flask by the addition of 1% (v/v) trypsin-EDTA (5 min in incubator). 10 mL of medium was added after trypsin incubation, to stop the enzymatic action. Cells were placed in a 15 mL tube and centrifuged at 300 rcf for 4 min. The supernatant was discarded and cells were washed with PBS (i.e. resuspended by pipetting and centrifuged again). The pellet was then resuspended in 2 mL of PBS and 2 mL of 4% PFA were added to fix the cells (20 min at 4^o^C). Cells were then washed with PBS. Nuclear staining was performed by using 1 mL of a 0.1 µg/ml DAPI solution (10 min at room temperature). Cells were washed with PBS and imaged on a glass slide.

NDs were produced with a second sonication with a total active time of 60 s at 72 W, with a pulse of 7 s and 30% duty cycle, and were stained with the fluorescent lipophilic dye DiI (molar ratio DSPC:PEG40s:DiI of 0.89:0.101:0.00025, 2.14 µM). NDs were incubated with cells for 40 min.

Cells were then pipetted onto a glass slide and a coverslip was placed above, to allow imaging with a fluorescence microscope (Axioimager M2m, Zeiss, Oberkochen, Germany).

1. **Illustration of NDs production protocol by sonication**


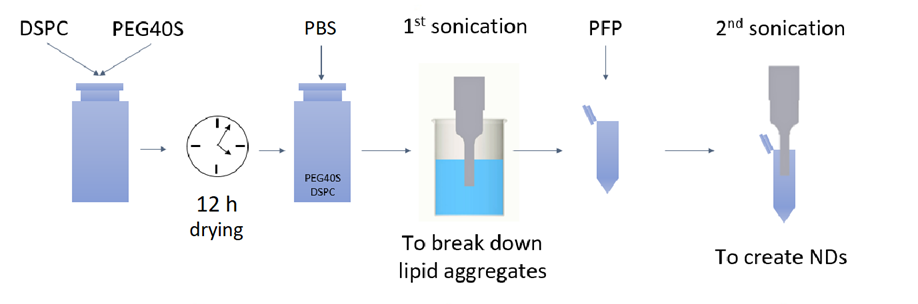


Figure D.1 Process of production of NDs. DSPC and PEG40s are dissolved in chloroform and placed in a glass vial; chloroform is then let to evaporate overnight. The lipid film is hydrated with PBS and a first sonication is performed with the tip completely immersed in the fluid, in order to break down lipid aggregates. PFP is then added to the lipid dispersion, and a second sonication is performed with the tip fully immersed in the liquid, to generate NDs.
